# Incorporating Liquid Crystal Display (LCD) Glass Waste as Supplementary Cementing Material (SCM) in Cement Mortars—Rationale Based on Hydration, Durability, and Pore Characteristics

**DOI:** 10.3390/ma11122538

**Published:** 2018-12-13

**Authors:** Seong Kyum Kim, Asad Hanif, Il Young Jang

**Affiliations:** 1 Research Institute for Mega Structures, Korea University, Seoul 02841, Korea; envylife@korea.ac.kr; 2 Department of Civil Engineering, Mirpur University of Science and Technology (MUST), Allama Iqbal Road Mirpur AJ & K 10250, Pakistan; ahanif@connect.ust.hk; 3 Department of Civil Engineering, Kumoh National Institute of Technology, Gumi 39177, Korea

**Keywords:** LCD, cement mortar, SCM, hydration characteristics, porosity

## Abstract

This paper assesses the feasibility of using liquid crystal display (LCD) waste glass as a supplementary cementing material in cement mortars. Two different sizes of LCD waste glass powder (LGP) particles were used (5 µm and 12 µm) with two substitution levels with cement in mortar (10% and 20%). The resulting mortars were evaluated for strength, hydration, porosity and durability through various experimental techniques. It was found that LGP particles lead to appreciable strength gain at all ages in comparison with control mortar, especially significant strength gain of 18% was observed at 28-day. This is attributed to the greater gel-space ratio as corroborated by the experimental determination of porosity, which is found less for LGP-incorporated mortars as compared to control cement mortar. The smaller particle size of LGPs not only accelerates the pozzolanic reaction in alkaline cementitious matrix, but also fills the smaller pores, thus reducing porosity and contributing to strength gain. Increased hydration was also elucidated qualitatively by backscattered electron imaging. Due to the increased hydration in LGP-incorporated pastes and mortars, the durability (in terms of chloride ion permeability) has also been found improved. Thus, it is established that 10% (by weight) of cement can be replaced with 12 μm LGP, whereas 20% can be replaced with 5 μm LDP for improved strength and durability. Incorporating LCD waste in mortars and concretes as partial replacement of cement can not only help utilize this potentially hazardous waste, but also significantly reduce the associated carbon dioxide emissions, thus promoting sustainable development.

## 1. Introduction

Concrete is the most widely used man-made material on earth. Its annual production is in billions of tons worldwide [1]. Cement, being the primary ingredient in concrete production, is, thus, produced in huge quantities, resulting in emissions of greenhouse gases to the environment. It is estimated that a metric ton of cement production results in emission of 931 kg of carbon dioxide in the environment [2,3]. Thus, from a sustainability point of view, cement consumption must be restricted either by using alternate binders such as magnesium phosphate cement [4], magnesium oxychloride cement [5], calcium sulfo-aluminate cement [6] and geopolymers [7,8], or incorporating pozzolanic materials as supplementary cementing material in concretes such as fly ash [9], silica fume [10], slag [11,12], metakaolin [13], rice husk ash [14], waste glass powder [15], etc. Another way is to incorporate different waste materials as aggregates in lieu of normal aggregates in concrete, like recycled aggregate concrete [3,16,17], ceramic waste [18,19,20], or crushed bricks [21]. Another potentially suitable material for use as supplementary cementing material (SCM) is liquid crystal display (LCD) glass waste, which has been investigated by a few researchers in recent years [22,23,24,25].

LCD waste glass is generated in tremendous amounts, owing to the development of the electronic display industry. With the increasing demand for handheld devices worldwide, LCD production is expected to grow exponentially, leading to increased LCD waste generation. While LCD glass is composed of silica, alumina and alkaline earth oxides, it differs largely from traditional glass [25]. LCD waste glass is usually divided into three classes: LCD cullet, LCD waste glass, and end-of-life LCD waste glass [26]. Such e-waste is potentially hazardous in many ways, owing to its non-degradability and toxic nature (due to its composition), thus creating hazards during disposal [27,28]. 

The current study focuses on LCD waste glass generated from LCD manufacturers due to defects in processing and cutting or bonding in the LCD panels. These defective LCD panels contain trace amounts of elements such as copper, manganese, molybdenum, and iron, and cannot be recycled due to extremely intricate recycling processes. Even if they are recycled, the resulting quality is degraded, making them unfit for customer use; thus, such panels are discarded in landfills. In 2015, around 40,000 tons of LCD glass panels were wasted in Korea alone [29]. 

Although a large number of studies on waste glass use as SCM have been conducted, research findings to substantiate LCD glass panel waste are sparse. The use of LCD waste glass as fine aggregate in concrete is well corroborated and it is shown that 20% sand can be replaced with LCD waste glass powder (LGP) [30,31]. It has also been shown earlier that LGP use as sand replacement can lead to improved durability [32] and better fire resistance [33]. A few studies conducted earlier also tried to use it as a supplement to binder. For instance, thin film transistor (TFT) LCD waste glass was used as SCM in concrete: 10% substitution was determined as optimal [34]. Further, TFT-LCD based binder pastes were formulated and the resulting hydration attributes were corroborated by thermal analyses [35]. It was also verified that cement-based composites (mortars and concretes), as well as ceramics can properly incorporate such waste without appreciable loss in strength and other properties [36].

Although the use of LCD glass panel waste as SCM in concrete and mortars has been studied, its detailed hydration attributes have not been investigated. This paper corroborates the use of LGP as partial replacement of cement in mortars by evaluating its strength, hydration, durability and porosity. A few studies published earlier have either only used LGP as a fine aggregate or have focused only on its compressive strength and hydration based on thermal analyses. This paper is novel because it investigates the durability of cement mortars, as well as innovatively corroborates the pozzolanic reaction of LGP in an alkaline cementitious matrix by using backscattered electron image analysis. 

## 2. Materials and Experimental Methods 

### 2.1. Materials Characterization

Ordinary Portland Cement (OPC) complying with KSL 5201 [37] was used as a primary binder in this experimental study. Standard quartz sand (fineness modulus; 2.92; water absorption; 2.40%, unit weight; 1597 kg/m^3^; specific gravity; 2.60, and 2.2% particles passing through #200 sieve) was used as fine aggregate in preparing the mortar specimens. The tests were conducted in accordance with Korean standards [38,39,40] and adopted in [41]. 

**Figure 1:**
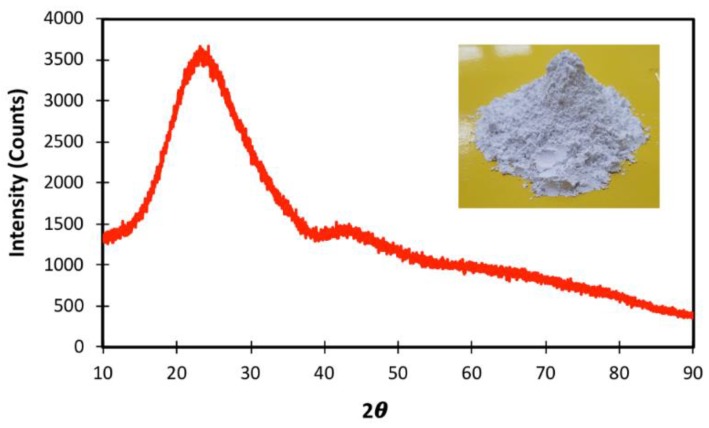
XRD of LGP particles.

**Figure 2:**
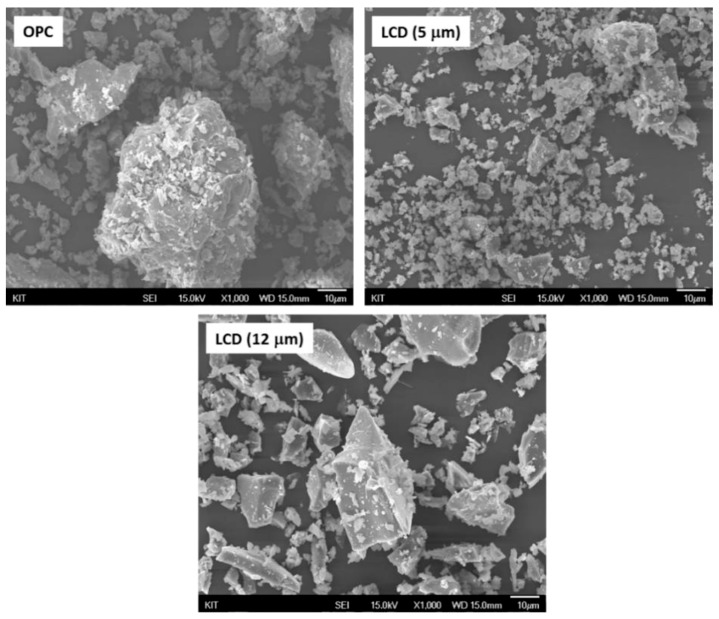
SEM images of raw material particles.

**Figure 3:**
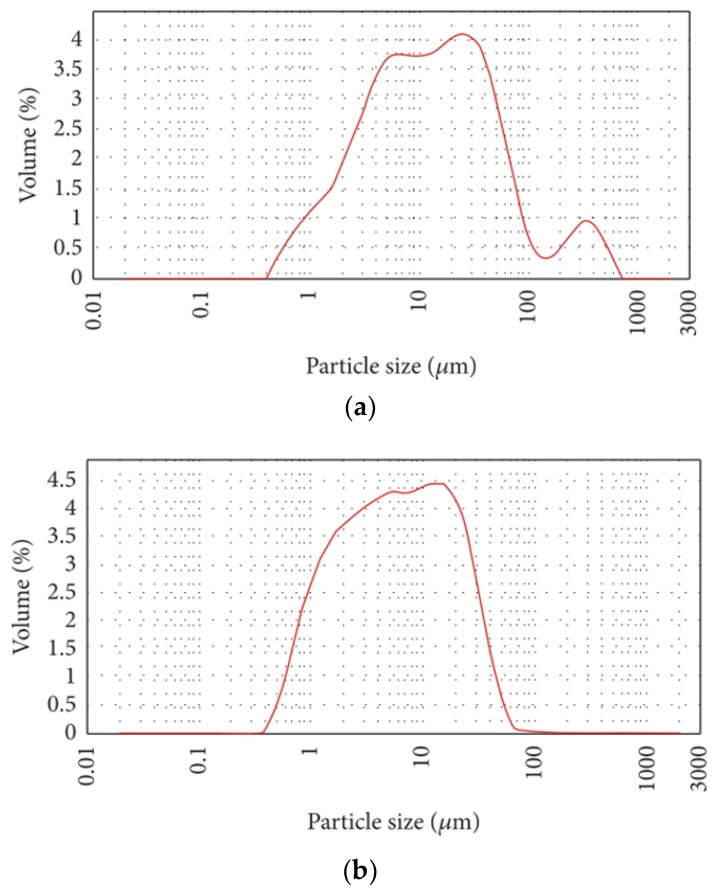
Particle size distribution (done by laser granulometry) of (**a**) LGP12, and (**b**) LGP5.

LCD glass waste was obtained from Inno. Co. Ltd., Daegu, Korea. Two different types of LGP were prepared by grinding the glass in ball mills (mean particle size 5 µm and 12 µm). The detailed material characterization results are shown in Figure 1, Figure 2 and Figure 3 and Table 1. The specific surface area of LCD particles was determined by the BET method [25] (Beckman Coulter SA3100 surface area and pore size analyzer, Brea, CA, USA). It can be seen from Table 1 that the mean particle size and surface area of LGP are comparable to OPC, and the chemical analysis indicates presence of both silica and lime. This hypothesizes the pozzolanic reaction of LGP in an alkaline cementitious matrix. However, the reactivity of LGP can be corroborated by X-Ray Diffraction (XRD), which is done on the Bruker advance-D8 power diffractometer (Billerica, MA, USA) with Cu-Ka radiation (λ = 0.154,178 nm) by subjecting the LGP particles to X-rays at a scanning rate of 10 °/min [26]. The broad hump in XRD diffractogram elucidates the amorphous nature of silica present in LGP; thus, it can react with calcium hydroxide and lead to formation of secondary hydrates [15,42,43].

### 2.2. Mixture Proportioning and Specimens Casting 

The objective of this study was to determine the suitability of LGP as SCM; therefore, it was substituted for cement in the mortars. Two substitution levels viz–a–viz 10% and 20% (by binder weight) were used. The substitution level was decided on the basis of available findings for other SCMs such as metakaolin, silica fume, etc., which have shown that any replacement level higher than 20% is detrimental to strength attributes of the hardened mortars and concretes [13,44]. The mix proportion (by weight) was selected as 1:0.4:2.13 binder (OPC + LGP), water, and sand. As the prime objective in this study was to determine the influence of LCD glass waste in water, the weight fraction of sand was not significant, as long as it was kept the same in all mix proportions [44]. The mixing regime comprised of dry mixing of all the powders and sand, followed by addition of required water and subsequent additional mixing until the achievement of a uniform consistent mix, observed through visual inspection. 50 mm side mortar cubes were cast for compressive strength testing and chloride penetration. In addition, pure binder paste samples (without sand) with a fixed water to binder ratio of 0.40 were also cast (cubes of 40 mm side) for evaluating the hydration attributes through backscattered electron imaging (BSE). The water-to-binder ratio for all the specimens were fixed at 0.40 to have a fair comparison of properties. The casted samples were covered with a polyethene sheet to prevent moisture loss, and subsequently demolded and put in a moist cabinet (at 25 °C temperature and 95% relative humidity) for curing until the desired testing age.

### 2.3. Experimental Methods and Procedures

#### 2.3.1. Compressive Strength Testing

Compressive strength was carried out on mortar cubes of 50 mm in accordance with American Society of Testing and Materials (ASTM) C109 [45]. The specimens were tested at various ages of 7 days, 28 days, and 56 days.

#### 2.3.2. Chloride Diffusion

The resistance to chloride ion penetration for LCD mortar with 28 days of curing was investigated in compliance with the Rapid Chloride Ion Penetration Test (RCPT) NT BUILD 443 [12,15,46,47] in which the specimens were exposed to 0.5 M NaCl. The increment of profile was 2 mm over 40 mm of penetration depth. By fitting the total chloride concentration profiles to the error function for chloride diffusion, regression parameters (i.e., surface chloride content and apparent chloride diffusion coefficient) were obtained:

(1) $$C\left(x,t\right)=C_{s}\left(1-erf\left(\frac{x}{\sqrt{4Dt}}\right)\right)$$

where ‘*C’* is the chloride concentration (% by sample weight), ‘*C*_s_’ is the surface chloride concentration (% by sample weight), *t* is the exposure time (sec), ‘*D’* is the apparent diffusion coefficient (m^2^/s). 

#### 2.3.3. Porosity and Pore Size Distribution

The porosity and pore volume characteristics of the pastes were studied by Mercury Intrusion Porosimetry (MIP, Micromeritics’ AutoPore IV 9500 Series, Norcross, GA, USA). Samples for MIP were taken from the fractured compressive strength specimens. Chunks of mortars were crushed to smaller pieces and pore water was removed by the solvent replacement method, with isopropanol as the solvent. The detailed experimental method is covered in [42,48,49,50,51,52]. 

#### 2.3.4. Backscattered Electron (BSE) Image Analysis

BSE was done on MIRA3 TESCAN (Brno-Kohoutovice, Czech Republic), SEM HV: 15 kV, Magnification: 500×, View field: 413 µm × 550 µm, Resolution: 0.5376 µm per pixel, WD: 10 mm, grayscale of BSE image: 0–255. For BSE, only the binder pastes were considered in order to determine the degree of hydration. The reason for choosing a binder paste as the test sample is to minimize the effects of aggregate inclusion, which may impose disruption in image processing due to silicate components in the aggregate. Thin sections of 5–10 mm were cut, and the free water was removed by immersing them in isopropanol. Then, the slices were saturated with resin, to support the microstructure, followed by polishing by abrasive papers of various grit sizes to achieve a smooth surface. The detailed polishing method, as used in the work of Hu and Li [53,54,55], was adopted. “The polishing of the sample is particularly critical for image analysis, as imperfections induced by polishing may cause problems during the segmentation of grains.” [56]. Later, the samples were carbon-coated for onward testing using an electron microscope. The samples were tested at the age of 7, 14, and 28 days. The porosity was determined by dividing the pore area by the total area, while the hydration degree was quantified by dividing the area of hydrated products by the total area of hydrated and un-hydrated products. The resulting values were shown in terms of corresponding percentages. The upper threshold grey level for porosity is determined from the inflection point of the cumulative brightness histogram of the BSE image. This represents a critical point where a small incremental grey value causes a sudden increase in threshold area, a condition termed as overflow [57]. The detailed methodological description is covered in great length in previously published literature [57,58].

## 3. Results, Discussion, and Analyses 

### 3.1. Compressive Strength

**Figure 4:**
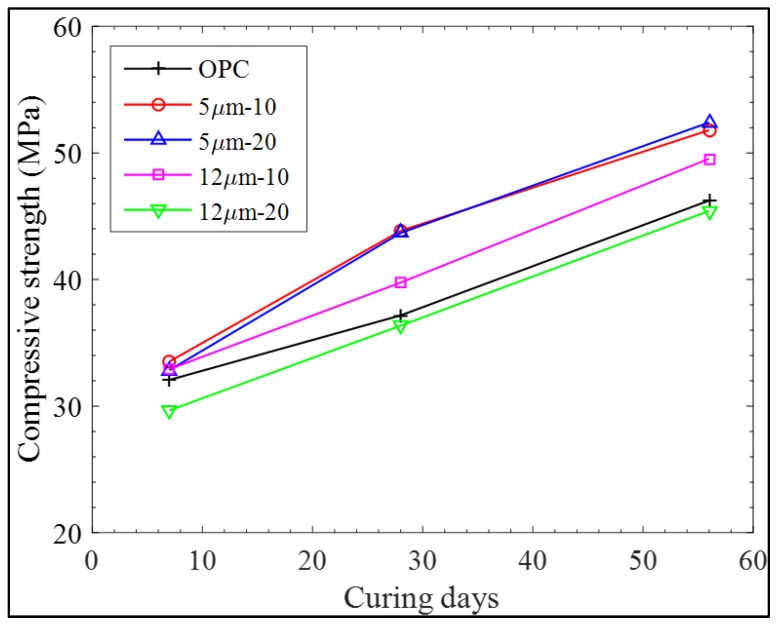
Compressive strength for LCD mortar at 7, 28, and 56 days.

The results for compressive strength are given in Figure 4. Typical behavior of cement-based composites of increasing strength with increase in curing age can be clearly seen. Further, the positive effect of LGP on strength enhancement is also obvious, particularly at 28 days, which is in agreement with the work of Kim et al. [37]. It is, however, important to point out the effect of particle size on the achieved strength.

The finer particles tend to involve more expressively in pozzolanic reactions [44,59], resulting in the formation of calcium silicate hydrate gel (reaction of calcium hydroxide present in the hydrated cement paste with the amorphous silica of LGP); their pore-filling effect is also well known [49,60]. Thus, in effect, the void ratio is reduced i.e., the gel-space is increased, resulting in increased compressive strength. At 7 days, age 5 μm LGP particles showed up to 4% strength increment in comparison with the control mortar (containing no LGP), whereas the 20 μm LGP particles showed a strength decline of 7.5% at the 20% replacement level, indicating their inertness and acting merely as fillers, though a meagre strength increase of 2% could be observed for 10% substitution level. At age of 28 days, optimum performance of LGPs could be seen, where LGP-5 μm resulted in a maximum strength increase of 18%. However, at 56 days, this increase was reduced to 13%. After this age, the hydration progresses so meagerly that it marginally affects the strength disparity [61], hence it can be safely said up to 13% strength can be improved with LGP addition with finer particle diameter.

### 3.2. Porosity and Pore Size Distribution

**Figure 5:**
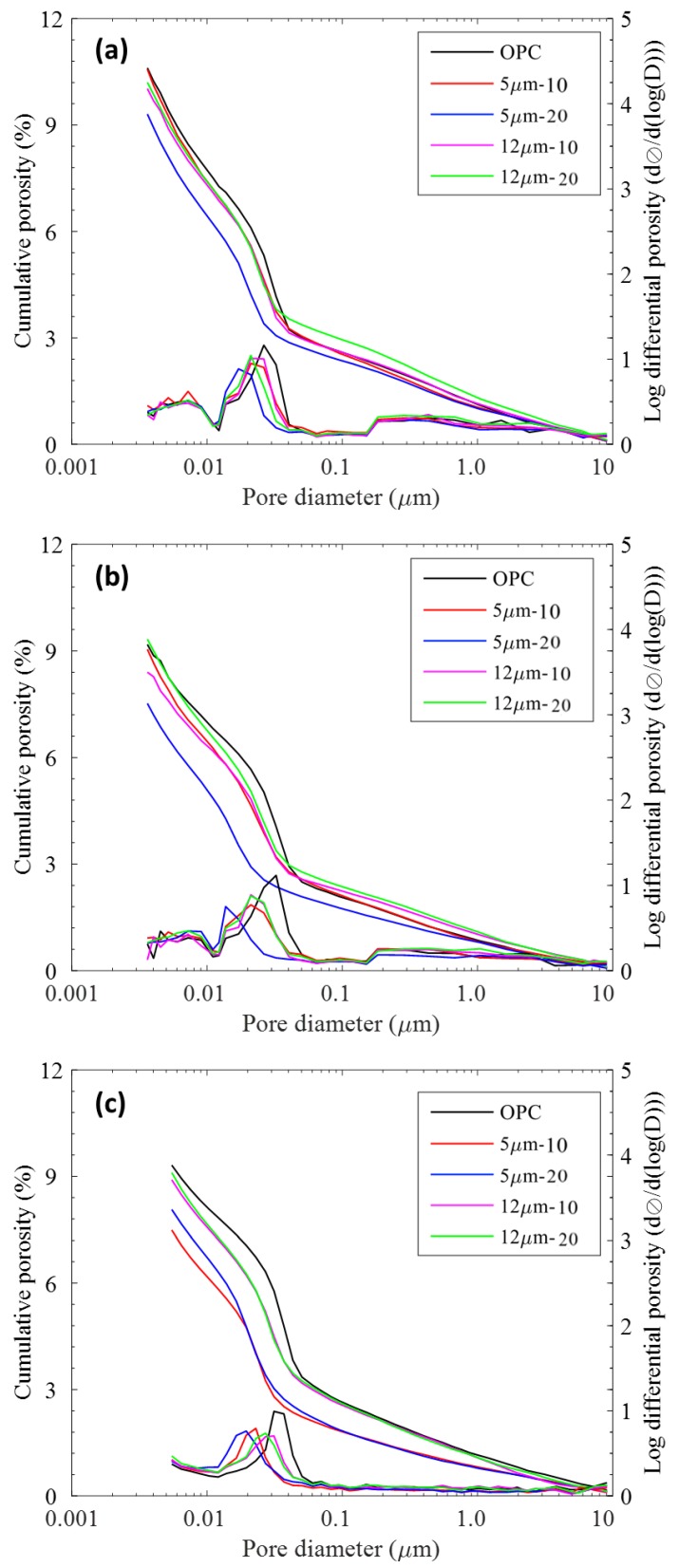
Cumulative porosity and log-differential porosity plots at (**a**) 7 days, (**b**) 14 days, and (**c**) 28 days.

In order to verify the increased gel-space ratio in LGP incorporated mortars, porosity was determined, which is directly correlated to gel-space ratio. The results of porosity and pore size distribution are plotted in Figure 5. The pore size distribution curves from MIP show that all pastes had a predominantly unimodal distribution (i.e., only one maxima peak). At age of 7 days, LGP incorporation did not influence porosity significantly. However, at later ages, it was clearly observed that LGP incorporation led to prominent decrease in porosity.

It is also pertinent to specify the importance of distribution of pores within the matrix. “Materials with even the same porosity can perform completely differently because of size and dispersal of pores, in the matrix” [49,52]. In the log differential plot (Figure 5; plotted on secondary y-axis), the critical pore diameter (d_c_) and threshold pore diameter (d_th_) tended to shift towards the left, which is the lower pore size range. d_c_ is calculated from the highest point of log differential plot, whereas d_th_ is identified from the slope of cumulative porosity plot. Substitution of cement with finer LGP particles (5 µm diameter) shifted these from a macro-pore zone to a meso-pore zone, resulting in finer pore structure. From the renowned strength-porosity relations [62], combined with the compressive strength results, the small differences are justifiable.

Bulk density and apparent (skeletal) density values were also obtained from MIP, which are reported in Table 2. LGP-incorporated mortars exhibited higher values of bulk and skeletal densities, except for LGP20, which signifies higher mechanical strength owing to a higher density of Calcium Slicate Hydrate (CSH) gel as compared to other hydration products [63]. This further substantiates that LGP incorporation leads to reduced air voids, thus improving mechanical properties.

### 3.3. Image Analysis for Porosity & Degree of Hydration

**Figure 6:**
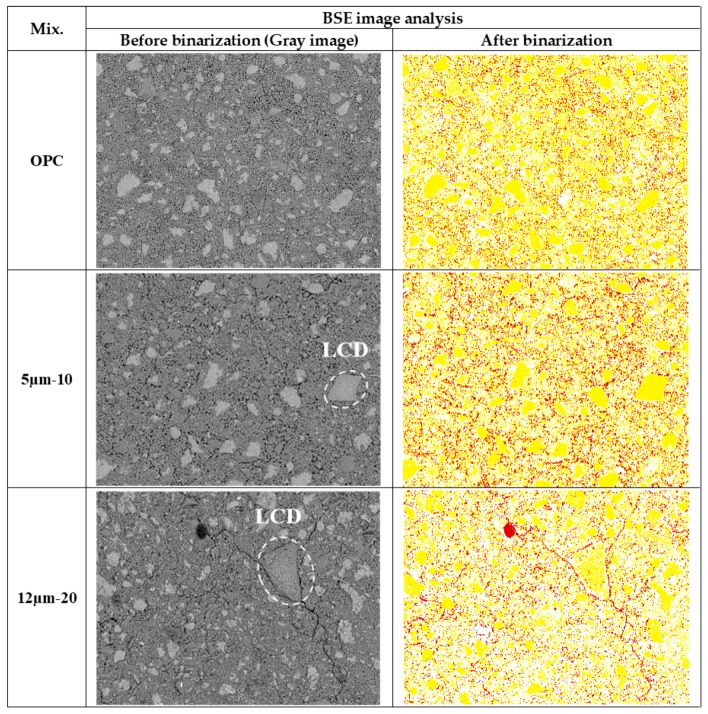
Example images before and after binarization for cement pastes at 14 days (pore: red, hydrated product: white, un-hydrated product: yellow).

**Figure 7:**
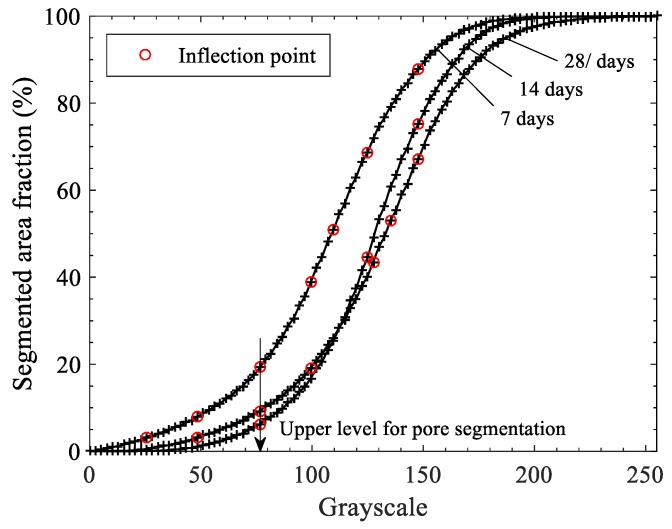
Determination of threshold level between pore and solid (OPC paste was used for the example).

The results of BSE analysis are given in Figure 6 and Figure 7 as well as tabulated in Table 3. The basic principle is that the volume fraction of a given phase is equal to the average surface fraction in a 2D microstructure, provided the number of sections analyzed is large enough to be statistically representative. Thresholding level between pore and solid is determined by finding the inflection point. The inflection point of the cumulative brightness histogram of a BSE image represents a critical transition where the segmented pore areas begin to ‘overflow’ to the surrounding paste [57]. All the curves shown in Figure 7 have a common value of inflection point (gray level 77), thereby used for the upper threshold level for all the paste samples. Thresholding levels between hydrated and un-hydrated products are 120, 135, and 148 respectively for 7, 14, and 28 days. With the specified threshold levels, degree of hydrations for OPC paste are 50.76%, 56.25%, 62.91%, respectively, for 7, 14, and 28 days. Those are equivalent to 51%, 56%, 62% at 0.4 w/c of OPC paste, respectively, for 7, 14, and 28 days [64]. It is to be clarified here that the results of porosity may differ in MIP and BSE because MIP was done on mortar specimens containing sand, whereas BSE was performed on binder pastes (justification is already presented earlier). 

For quantifying the degree of hydration, other methods such as XRD with Rietveld analysis, or Thermogravimetric Analysis (TGA) may be used which are faster as well as more accurate (the relative error on degree of hydration is about 5–10% by image analysis in comparison with only 2–3% by XRD-Rietveld analysis [58]). However, BSE offers better understanding of microstructural attributes like phase morphology, phase distribution, C-S-H density, and porosity.

### 3.4. Chloride Diffusion 

**Figure 8:**
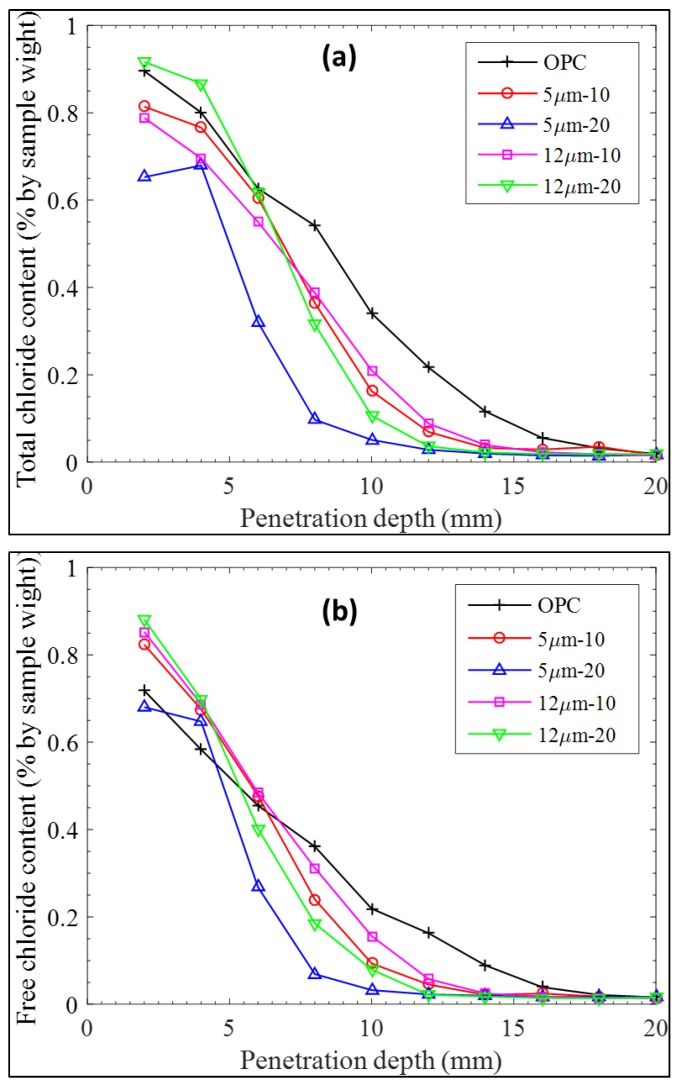
Concentration profiles for (**a**) free chloride, and (**b**) total chloride.

The results of chloride diffusion after 28 days are represented in Figure 8 and Table 4. It was seen that 20% addition of LGP12 is detrimental to the mortars, as it increases the chloride content in the specimens after exposure to chloride salts. The 10% addition of either LGP (finer or coarser) led to improved durability properties indicated by the lower chloride content residing in the specimens at various depths, while 20% addition of LGP5 resulted in optimum results. Thus, it is quite evident from RCPT that the addition of LGP in cement mortars lead to better durability due to greater packing of solid phases in the matrix.

## 4. Inferences, Conclusions, and Prospects—A Way Forward

In this study, the feasibility of LCD waste glass as partial replacement of cement was assessed through strength, hydration, and durability characteristics. LCD waste glass was milled and ground to finer particle sizes. Two types of LGP were used: one, with mean particle size of 5 μm, and other, with mean particle size of 12 μm. Mortars and binder paste specimens were cast keeping the fixed water to binder (cement plus LGP) ratio of 0.40, while two replacement levels of 10% and 20% were employed.

The following conclusions and inferences are drawn from the experimental results and analyses:

(1) LGP is useful in improving the hydration degree and hydration rate at almost all the curing ages; however, the beneficial effect is more pronounced at later stages of strength development (28 days).
(2) The particle size and surface area of LGP greatly affect the pozzolanic reaction in alkaline cementitious matrix. Thus, finer particles increase the reaction rate and help in strength gain in a more effective way than coarser particles. More refined, compact, and uniform microstructural features are also prominent in case of LGP-incorporated pastes.
(3) LGP addition in cement mortars and pastes significantly improves strength and durability owing to reduced porosity, primarily due to the reaction of amorphous silica (present in LGP) with calcium hydroxide (cement hydration product), resulting in the formation of secondary hydrates, and secondarily due to the pore filling effect indebted to the fine LGP particle size.

Although the substitution of cement with LGP leads to improvement in mechanical, durability, and microstructural properties, the deterministic characteristics of LGP particles include particle size/surface area as well as the substitution amount. Up to 20% (by weight) of cement can be replaced with finer LGP particles (size up to 5 µm), whereas 10% cement replacement by coarser LGP particles (size up to 12 µm) is deemed useful. This can substantially reduce the cement consumption in mortars and concrete, leading to reduced carbon dioxide emissions and a cleaner environment. For typical building construction and rendering or masonry, the LGP addition is definitely not harmful. However, for certain other specific uses of concretes and mortars, such as radiation shielding, thermally conductive composites for use in structural health monitoring, and lightweight concrete must be carefully studied. Another important study area is the behavior of LGP particles in the presence of nano-materials in cementitious composites: Would the reactivity of LGP increase in the presence of nano-materials? Or would the likelihood of agglomeration of nano-materials inhibit the pozzolanic activity of LGP, and likewise? Such studies need to be further carried out for its deliberation as a permanent cement replacement solution.

## Figures and Tables

**Table 1:** Physical and chemical properties of raw materials used in the study.

Binder	Mean Size (µm)	Surface Area (m^2^/g)	Oxide Composition (%)						
			MgO	Al_2_O_3_	SiO_2_	CaO	Fe_2_O_3_	SO_3_	K_2_O
OPC	21	1.288	2.84	4.04	19.0	65.1	3.09	3.77	1.570
LCD	5	0.996	0.4	18.2	66.9	10.0	0.08	-	0.057
	12	0.935	0.42	18.0	65.7	10.3	0.14	-	0.044

**Table 2:** Bulk and skeletal densities obtained through MIP.

Mix ID	7-Day Age		14-Day Age		28-Day Age	
	Bulk Density (g/cc)	Skeletal Density (g/cc)	Bulk Density (g/cc)	Skeletal Density (g/cc)	Bulk Density (g/cc)	Skeletal Density (g/cc)
OPC	2.123	2.475	2.159	2.453	2.120	2.425
5 µm-10	2.130	2.470	2.129	2.426	2.159	2.406
5 µm-20	2.087	2.382	2.094	2.344	2.152	2.411
12 µm-10	2.131	2.463	2.180	2.461	2.136	2.417
12 µm-20	2.059	2.392	2.070	2.360	2.109	2.393

**Table 3:** Parameters obtained from BSE image analysis for cement pastes at various ages (* the values indicate the average of porosity and hydration degree calculated from various frames in BSE imaging).

Mix	Parameter	Average Values*		
		7 Days (Age)	14 Days (Age)	28 Day (Age)
**OPC**	Porosity (%)	19.31	6.37	9.88
	Hydration Degree (%)	50.76	56.25	62.91
**5 μm-10**	Porosity (%)	20.51	11.61	7.22
	Degree of Hydration (%)	43.76	61.72	67.11
**5 μm-20**	Porosity (%)	21.70	10.54	8.16
	Hydration Degree (%)	39.63	52.45	65.96
**12 μm-10**	Porosity (%)	22.36	7.57	6.51
	Hydration Degree (%)	42.42	60.65	69.04
**12 μm-20**	Porosity (%)	18.73	12.37	13.16
	Hydration Degree (%)	35.27	67.55	83.66

**Table 4:** Diffusion parameters obtained from the total chloride concentration profile.

Mix	*C*s (% by Sample Weight)	*D* (E-12 m^2^/s)	R Squared
OPC	1.15	12.28	0.98
5 μm-10	1.14	8.24	0.96
5 μm-20	1.01	4.86	0.93
12 μm-10	1.06	8.98	0.97
12 μm-20	1.33	6.59	0.95
